# Development and validation of a prognostic prediction model for patients with traumatic multiple fractures and hemorrhagic shock using an Automated Machine Learning framework: a retrospective cohort study

**DOI:** 10.3389/fmed.2026.1837872

**Published:** 2026-06-10

**Authors:** Tingyong Han, Yan Li, Xuewei Mu, Peng Wu, Zhigang Zhang, Liangjie Zhang, Zhifei Liang, Long Li

**Affiliations:** 1 Department of Emergency, Ya’an Polytechnic College Affiliated Hospital, Ya’an, Sichuan, China; 2 Faculty of Clinical Medicine, Ya’an Polytechnic College, Ya’an, Sichuan, China; 3 Department of Emergency, Ya’an People's Hospital, Ya’an, Sichuan, China; 4 Department of Emergency, Yucheng District People’s Hospital of Ya’an, Ya’an, Sichuan, China; 5 Department of Emergency, Mingshan District People’s Hospital of Ya’an, Ya’an, Sichuan, China; 6 Department of Emergency, Ya’an Hospital of Traditional Chinese Medicine, Ya’an, Sichuan, China; 7 Department of Surgery, Ya’an Renkang Hospital, Ya’an, Sichuan, China; 8 Department of Emergency and Critical Care Medicine, The 945th Hospital of the Joint Logistics Support Force of the Chinese People’s Liberation Army, Ya’an, Sichuan, China

**Keywords:** Automated Machine Learning, mortality, prediction model, prognosis, traumatic multiple fractures with hemorrhagic shock

## Abstract

**Objective:**

To develop and validate a prognostic prediction model for patients with traumatic multiple fractures and hemorrhagic shock using an Automated Machine Learning (AutoML) framework, evaluating its predictive performance and clinical utility.

**Methods:**

A total of 1,028 patients with traumatic multiple fractures and hemorrhagic shock admitted to the Emergency Departments and Intensive Care Units of seven public hospitals between January 2020 and December 2025 were retrospectively enrolled, with data from 4 hospitals designated as the training set (*n* = 720) and data from 3 hospitals serving as the test set (*n* = 308). Multidimensional data—including demographic characteristics, trauma/injury features, admission vitals/perfusion indices, and laboratory parameters—were extracted. The improved beaver behavior optimizer (IBBO) algorithm synchronously optimized feature subsets, base learners, and hyperparameter combinations. Clinical rationality of features was verified using LASSO regression and SHAP interpretability analysis.

**Results:**

The IBBO algorithm demonstrated superior stability and outperformed the original BBO and comparative algorithms in most test functions. The AutoML model achieved best performance. The test set further confirmed its robustness, yielding a ROC-AUC of 0.9357 and PR-AUC of 0.9270. Decision curve analysis demonstrated that the AutoML model’s clinical net benefit surpassed that of traditional methods across a threshold range of 1–96%; The calibration curve likewise indicated high consistency between predicted probabilities and actual outcomes, with a Brier score as low as 0.110. SHAP analysis identified key predictors in descending order of importance: GCS score, ISS score, time from injury to ER admission, lactate, and fibrinogen.

**Conclusion:**

The IBBO-based AutoML prognostic model provides an efficient, accurate tool for in-hospital mortality prediction in traumatic multiple fractures with hemorrhagic shock. The model identified that core predictors—including GCS score, ISS score, time to ER admission, lactate, and fibrinogen—critically influence in-hospital mortality outcomes. Clinical decision support software derived from this model offers visual, intelligent guidance for stratified care, promising utility in trauma emergency practice.

## 1 Introduction

Traumatic multiple fractures complicated by hemorrhagic shock represent critical emergencies in emergency surgery and intensive care medicine. This condition is characterized by a sudden reduction in effective circulating blood volume, inadequate systemic tissue perfusion, and multi-organ dysfunction, often accompanied by severe inflammatory responses and coagulation disorders, leading to rapid clinical deterioration, frequent complications, and high in-hospital mortality (1). In recent years, although advancements in the trauma care system have improved survival rates among these patients, a subset continues to face high mortality risks due to factors such as injury severity, baseline physiological status, and timeliness of intervention, making this a pivotal challenge in trauma resuscitation (2). Consequently, accurate prognostic assessment for patients with traumatic multiple fractures and hemorrhagic shock is crucial for individualized risk stratification and targeted treatment, thereby reducing mortality (3). Traditional prognostic evaluation methods for trauma patients predominantly rely on clinicians’ empirical judgment or assessments based on singular injury scores or vital signs, such as the Injury Severity Score (ISS), Glasgow Coma Scale (GCS), and systolic blood pressure (4). Although straightforward to implement, these approaches suffer from limitations including unidimensional assessment, inadequate consideration of multifactorial interactions, and suboptimal sensitivity/specificity. Such shortcomings may fail to comprehensively reflect patients’ pathophysiological states, leading to prognostic inaccuracies that compromise the scientific validity and timeliness of therapeutic decisions (5, 6). As clinical research evolves, mounting evidence indicates that the prognosis of traumatic multiple fractures with hemorrhagic shock involves synergistic influences from demographic characteristics, trauma features, vital signs, and laboratory parameters. These exhibit intricate nonlinear correlations and interactions, rendering precise prediction unattainable through isolated or limited indicators (7, 8).

Automated Machine Learning (AutoML), an innovative approach in the machine learning domain, leverages automated workflows for feature engineering, model selection, hyperparameter tuning, and other modeling steps (9). This minimizes subjective human intervention, uncovers latent associations within multidimensional clinical data, and significantly enhances prediction performance and generalizability (10). Compared to traditional machine learning, AutoML adapts better to clinical datasets with high dimensionality, complexity, and class imbalance, demonstrating strong potential in prognostic predictions for oncology, cardiovascular diseases, and critical care (11, 12). Concurrently, advances in intelligent optimization algorithms, such as the improved beaver behavior optimizer (IBBO) (13), elevate AutoML’s capabilities. These metaheuristic algorithms enhance stability and predictive efficiency by optimizing population distribution and balancing exploration-exploitation trade-offs, thereby refining feature selection and hyperparameter optimization (14, 15). However, studies applying IBBO-based AutoML to prognostic prediction in traumatic multiple fractures with hemorrhagic shock remain scarce, with limited exploration of model interpretability and clinical translation pathways.

Against this backdrop, this study integrates multidimensional clinical data to construct a prognostic prediction model for patients with traumatic multiple fractures and hemorrhagic shock using AutoML. We aim to provide a precise, efficient prognostic tool and scientific basis for individualized, stratified clinical management.

## 2 Methods

### 2.1 Study subjects and data collection

This multicenter retrospective cohort study enrolled patients from seven major medical institutions in Ya’an: Ya’an Polytechnic College Affiliated Hospital (*n* = 211), PLA 945 Hospital (*n* = 75), Ya’an People’s Hospital (*n* = 315), Yucheng District People’s Hospital of Ya’an (*n* = 119), Ya’an Hospital of Traditional Chinese Medicine (*n* = 121), Mingshan District People’s Hospital of Ya’an (*n* = 135), and Ya’an Renkang Hospital (*n* = 52). A total of 1,028 patients with traumatic multiple fractures and hemorrhagic shock admitted between January 2020 and December 2025 met the inclusion/exclusion criteria. As a retrospective study, informed consent was waived. Ethical approval was obtained from all institutional ethics committees in compliance with the Declaration of Helsinki.

Inclusion criteria: ① Age ≥18 years; ② radiologically confirmed traumatic multiple fractures; ③ diagnosis of hemorrhagic shock at admission (systolic blood pressure <90 mmHg or >40 mmHg drop from baseline, with clinical signs of hypoperfusion, e.g., elevated lactate, altered consciousness); ④ complete clinical data.

Exclusion criteria: ① prehospital death or discharge/withdrawal within 24 h; ② comorbid end-stage diseases (e.g., advanced malignancy, severe cirrhosis, uremic chronic renal failure); ③ missing key predictive variables or outcomes.

Based on trauma resuscitation guidelines, critical care pathophysiology, and literature, multidimensional variables potentially linked to prognosis were systematically collected. All variables were operationally defined, covering: (1) Demographics and baseline characteristics: Age, sex, body mass index (BMI), comorbidities (hypertension, diabetes, coronary heart disease, chronic obstructive pulmonary disease). (2) Trauma and injury characteristics: Injury mechanism (traffic accident, fall, crush injury), time from injury to ER admission, traumatic brain injury (CT-confirmed), abdominal solid organ injury, Injury Severity Score (ISS), Glasgow Coma Scale (GCS). (3) Admission vital signs and perfusion indices: Initial vital signs (systolic/diastolic pressure, heart rate, respiratory rate, temperature); shock index (heart rate/systolic pressure). (4) Laboratory parameters: first venous or arterial blood gas within 6 h post-admission (or pre-resuscitation), including: ① hematology and coagulation: Hemoglobin (Hb), hematocrit (HCT), platelet count (PLT), fibrinogen (Fib), activated partial thromboplastin time (APTT), prothrombin time (PT), international normalized ratio (INR); ② metabolic and perfusion markers: Arterial lactate (Lac), base excess (BE); ③ organ function and electrolytes: Creatinine (Cr), blood urea nitrogen (BUN), alanine aminotransferase (ALT), sodium, potassium. Two blinded researchers independently extracted data using standardized case reports, with inter-rater reliability >0.85 (Kappa); discrepancies were resolved by a senior clinician. Missing data were handled based on variable type and missing proportion, with only a few variables exhibiting random missingness below 5%; detailed information is provided in the variable summary table (Supplementary Table S1). Multiple imputation using chained equations with five iterations was applied to continuous variables, whereas for categorical variables, imputation was performed directly using the mode.

The primary outcome was in-hospital all-cause mortality. Data splitting by center rather than by time or random assignment, data from Affiliated Hospital of Ya’an Polytechnic College Affiliated Hospital, PLA 945 Hospital, Ya’an People’s Hospital, and Yucheng District People’s Hospital formed the training set (*n* = 720) for AutoML development. Data from Mingshan District People’s Hospital, Ya’an Hospital of Traditional Chinese Medicine, and Ya’an Renkang Hospital constituted the test set (*n* = 308) for validation.

### 2.2 Automated Machine Learning model construction

An AutoML framework integrating IBBO was proposed to synchronize feature selection and hyperparameter tuning. Inspired by natural beaver dam-building behaviors, beaver behavior optimizer (BBO) is a metaheuristic algorithm emphasizing social cooperation and adaptive planning (13). IBBO was enhanced via chaotic mapping (initial population distribution) and dynamic Lévy flight (exploration-exploitation balance), validated using CEC2022 benchmarks.

**Figure 1:**
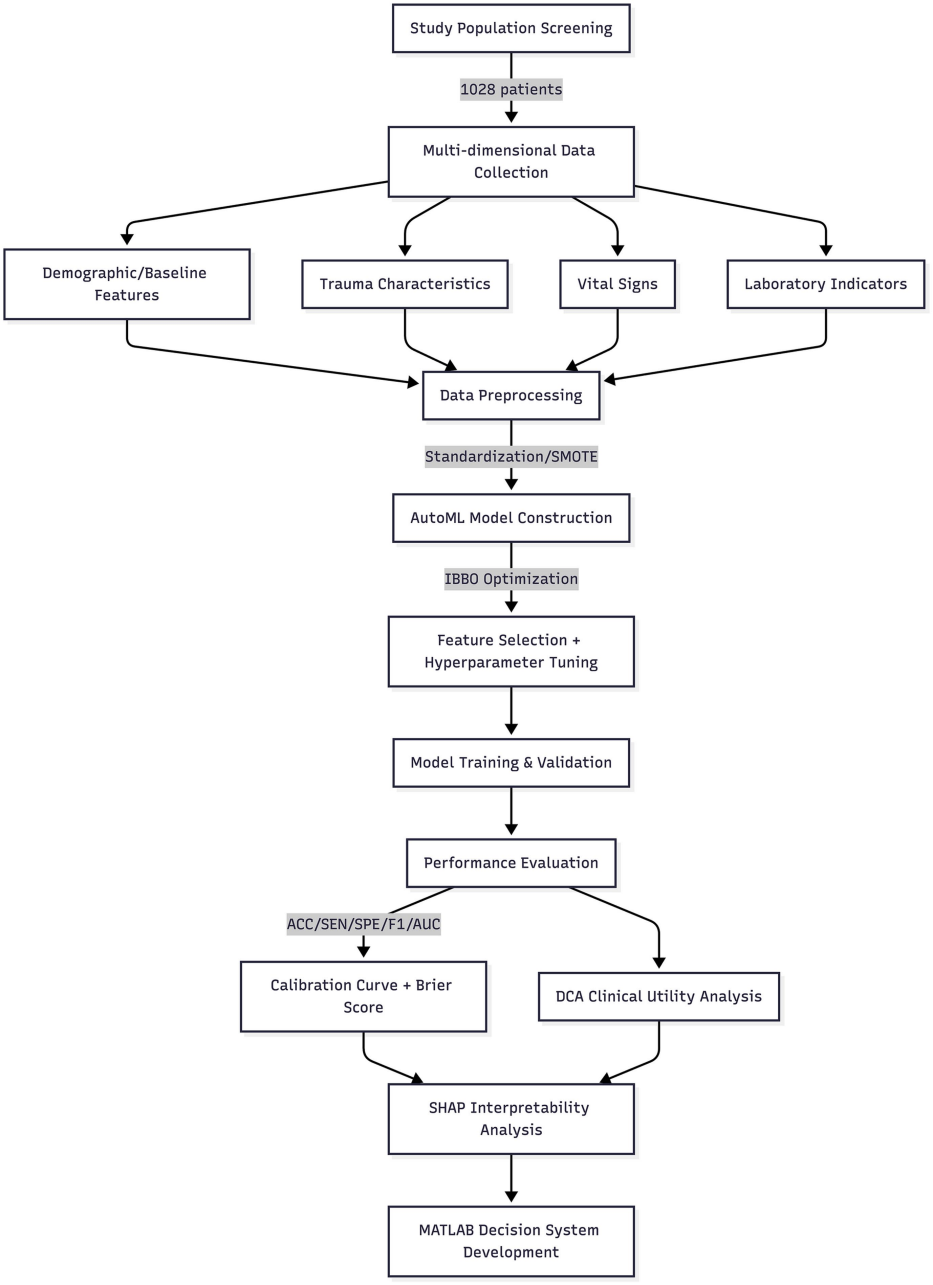
Technical workflow diagram.

A two-phase optimization design was implemented: phase I filtered high-weight feature subsets in discrete space; Phase II fine-tuned hyperparameters in continuous space. Six models were compared: Logistic Regression (LR) (16), Support Vector Machine (SVM) (17), Adaptive Boosting (AdaBoost) (18), Extreme Gradient Boosting (XGBoost) (19), LightGBM (20), and the proposed AutoML. All models were implemented in MATLAB 2024b. The training set used five-fold cross-validation. Given that the in-hospital mortality rate was 31%, presenting a clear class imbalance that could bias model training, this study applied the Synthetic Minority Oversampling Technique (SMOTE) to rebalance only the training set. By generating artificial minority class samples in the training set, the ratio of death to survival cases was made 1:1, while the independent test set completely retained the original imbalanced distribution to ensure objectivity in model performance evaluation. To verify the actual impact of the SMOTE resampling strategy on model performance, we systematically compared predictive performance with and without SMOTE using the AutoML model as a benchmark in the Results section (see Supplementary Table S2). See Figure 1 for technical workflow.

### 2.3 Evaluation metrics

This study established a multidimensional model evaluation framework. For classification performance, the following core metrics were comprehensively adopted to evaluate the model’s discrimination ability for in-hospital mortality prediction: Accuracy (ACC), Sensitivity (SEN), Specificity (SPE), Positive Predictive Value (PPV), Negative Predictive Value (NPV), F1-score (harmonic mean of precision and recall), Area Under the Receiver Operating Characteristic Curve (ROC-AUC), and Area Under the Precision-Recall Curve (PR-AUC). Under class imbalance, this indicator system can systematically evaluate the model’s discrimination ability and predictive stability. Calibration performance was evaluated using calibration curves and the Brier score (lower Brier scores indicate higher predictive accuracy). Clinical utility was quantified using Decision Curve Analysis (DCA), calculating the Net Benefit across different risk thresholds, to validate the model’s effective clinical decision-making range.

### 2.4 Interpretability analysis

After initial feature selection via the AutoML framework, LASSO regression was applied to validate the robustness of the selected features (21). Subsequently, the SHapley Additive exPlanations (SHAP) model was used to analyze feature clinical plausibility. The specific workflow comprised: (1) AutoML feature pre-screening: Utilizing predefined search spaces and optimization objectives, the AutoML algorithm automatically identified the feature subset most significantly associated with prognosis; (2) LASSO feature validation: LASSO regression was applied to the AutoML-selected subset. Its sparsity and stability were verified through the regularization constraint mechanism, ensuring feature resistance to overfitting. Differences between LASSO-selected and AutoML-selected features were compared; (3) SHAP interpretability analysis: By calculating Shapley values for each feature, quantitative attribution of feature contributions to individual predictions was achieved; summary plots were comprehensively used to display global feature importance rankings, while waterfall plots, decision path plots, and force plots were employed to visually illustrate the interpretation process of specific prediction cases, thereby systematically revealing the model’s intrinsic decision-making logic.

### 2.5 Interactive tool development

For clinical implementation, the App Designer module in MATLAB 2024a was used to develop a Clinical Decision Support software tool. This software integrates the final optimized machine learning model to provide clinicians with an intuitive, user-friendly interface for assessing patient transfusion requirements.

### 2.6 Statistical analysis

All data were uniformly imported into the SPSS 26.0 statistical platform for processing. The normality of continuous variables was assessed using the Shapiro–Wilk test, with *p* > 0.05 considered indicative of an approximately normal distribution. Continuous variables conforming to a normal distribution are presented as mean ± standard deviation (*x̄* ± *s*), while non-normally distributed continuous variables are presented as median with interquartile range [*M* (Q1, Q3)]; categorical variables are presented as frequency and percentage (*n* %). For inter-group comparisons, an independent samples *t*-test was used for normally distributed continuous variables, while the Mann–Whitney *U* test was applied for non-normally distributed ones; Pearson’s chi-square test was employed for categorical variable comparisons. All statistical analyses adopted two-tailed tests, with *p* < 0.05 serving as the threshold for statistical significance. The results are presented in standardized tabular format.

## 3 Results

### 3.1 Characteristics of study subjects

The overall cohort had a mean age of 56.14 ± 13.85 years, with 735 males (71.50%) and 293 females (28.50%). No significant differences existed between the training set (*n* = 720) and test set (*n* = 308) across all baseline characteristics (all *p* > 0.05), confirming effective stratified randomization. In-hospital mortality rates were consistent (training set: 30.97% vs. test set: 33.44%; χ^2^ = 0.607, *p* = 0.436). Details are summarized in Table 1.

**Table 1:** Baseline characteristics of the study subjects.

Features	Training set (*n* = 720)	Testing set (*n* = 308)	Statistic	*p*-value
Outcome				
In-hospital mortality, *n* (%)	223 (30.97)	103 (33.44)	0.607	0.436
Demographic				
Age (years), Mean ± SD	56.24 ± 13.78	55.91 ± 14.02	0.350	0.726
Male, *n* (%)	520 (72.22)	215 (69.81)	0.618	0.432
BMI (kg/m^2^), Mean ± SD	23.45 ± 3.67	23.21 ± 3.82	0.949	0.343
Comorbidities, *n* (%)	245 (34.03)	111 (36.04)	0.385	0.535
Trauma and injury characteristics				
Traffic injury mechanism, *n* (%)	405 (56.25)	168 (54.55)	0.254	0.614
Injury-to-ER time >4 h, *n* (%)	251 (34.86)	112 (36.36)	0.213	0.644
Concomitant traumatic brain injury, *n* (%)	288 (40.00)	129 (41.88)	0.317	0.573
Concomitant abdominal visceral injury, *n* (%)	187 (25.97)	84 (27.27)	0.188	0.665
GCS score, *M* [Q1, Q3]	10 [6, 14]	10 [6, 14]	0.452	0.651
ISS score, *M* [Q1, Q3]	30 [25, 36]	29 [25, 35]	0.827	0.408
Vital signs and perfusion indicators				
Systolic blood pressure (mmHg), Mean ± SD	89.34 ± 12.45	88.97 ± 13.01	0.431	0.667
Diastolic blood pressure (mmHg), Mean ± SD	56.78 ± 10.23	57.12 ± 10.45	0.485	0.628
Heart rate (beats/min), Mean ± SD	112.36 ± 18.23	114.02 ± 17.89	1.353	0.176
Shock index, *M* [Q1, Q3]	1.26 [1.05, 1.52]	1.28 [1.07, 1.55]	1.104	0.270
Laboratory indicators				
Hemoglobin (g/L), Mean ± SD	78.91 ± 10.34	79.45 ± 9.87	0.777	0.437
Platelet count (×10^9^/L), M[Q1, Q3]	165 [125, 205]	170 [128, 208]	0.785	0.433
Lac (mmol/L), M[Q1, Q3]	4.1 [2.8, 6.0]	4.0 [2.7, 5.9]	0.594	0.553
Fib (g/L), Mean ± SD	3.45 ± 0.98	3.38 ± 1.02	1.036	0.300
APTT (s), Mean ± SD	37.85 ± 4.12	38.20 ± 4.33	1.229	0.219
PT (s), Mean ± SD	15.62 ± 2.45	15.78 ± 2.61	0.940	0.347
Cr (μmol/L), *M* [Q1, Q3]	88 [72, 110]	85 [70, 108]	1.022	0.307

Continuous variables conforming to a normal distribution are presented as Mean ± SD, otherwise as M[Q1, Q3]. Categorical variables are presented as *n* (%).

### 3.2 Simulation testing for algorithm improvement

**Figure 2:**
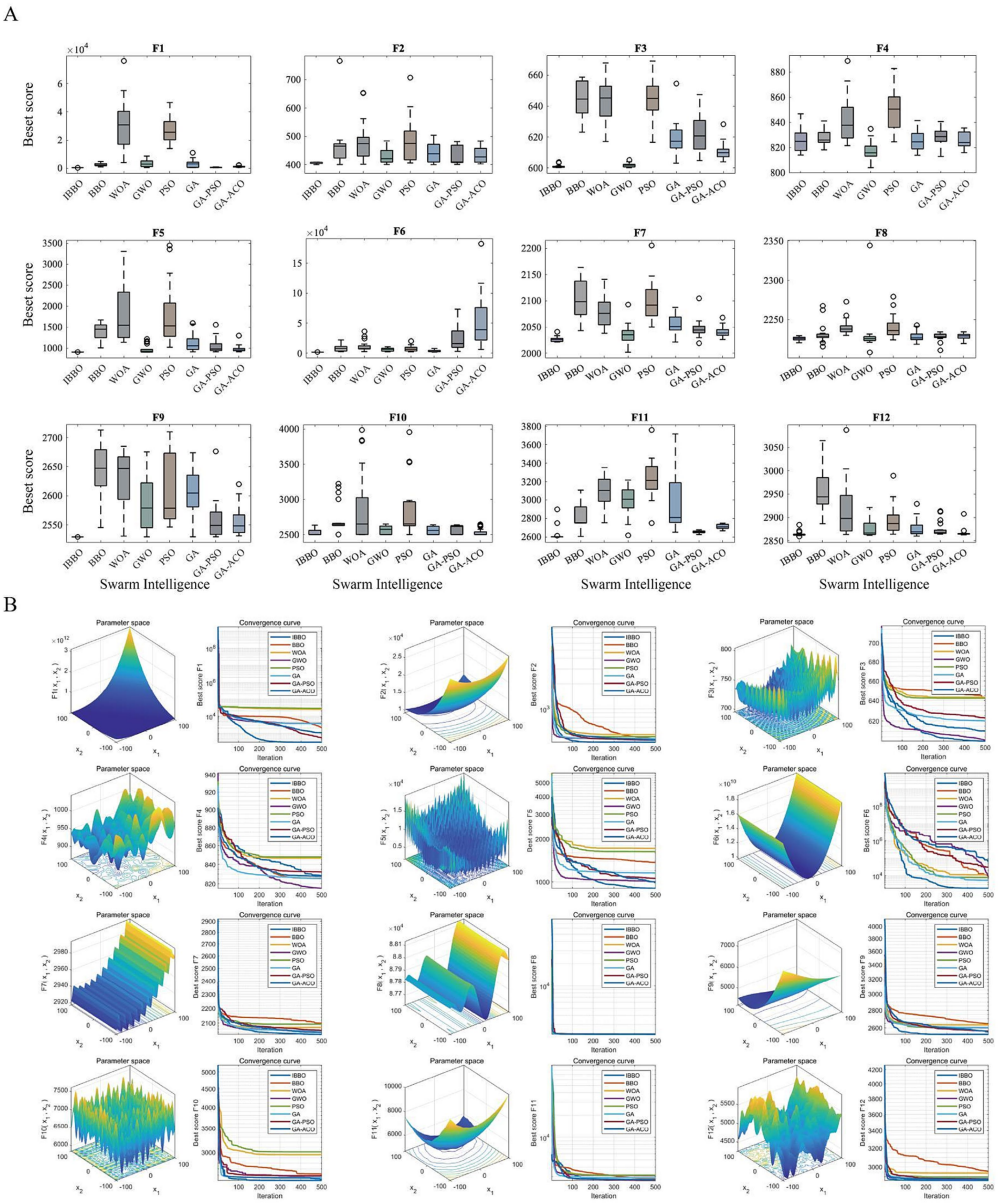
Simulation tests for improved swarm intelligence algorithms. **(A)** Box plots of optimization results across 30 runs on CEC2022 benchmark functions, illustrating stability and robustness; **(B)** convergence curves during optimization, reflecting speed and ability to escape local optima.

To validate the optimization capability of the IBBO algorithm, comparative tests were conducted against original BBO, Whale Optimization Algorithm (WOA), Grey Wolf Optimizer (GWO), Particle Swarm Optimization (PSO), Genetic Algorithm (GA), GA-PSO, and GA-ACO. Experiments utilized all 12 benchmark functions from the CEC2022 suite, with dimension = 10, population size = 30, maximum iterations = 500, and 30 independent runs for statistical reliability. Box plots from 30 runs demonstrated IBBO’s superior stability over others (Figure 2A). Convergence curves further revealed IBBO’s faster convergence and reduced risk of local optima (Figure 2B). These results validate IBBO’s global optimization efficiency.

### 3.3 Model training and performance evaluation

Given the approximately 31% incidence of in-hospital mortality events in the training set, the SMOTE was applied for rebalancing, and all data were incorporated into five-fold stratified cross-validation. Within the AutoML framework, the improved IBBO algorithm executed two-stage synchronous optimization: the first stage searched for the optimal subset among 34 candidate features, while the second stage synchronously selected the base learner and optimized its hyperparameters. The final prediction model was composed of five core features—Glasgow Coma Scale (GCS), Injury Severity Score (ISS), time from injury to emergency room admission, lactate (Lac), and fibrinogen (Fib)—with LightGBM selected as the base learner. The optimal hyperparameters were determined as: maximum depth of 7, learning rate of 0.08, minimum number of samples per leaf node of 15, L2 regularization coefficient of 0.1, and column sampling rate of 0.8. Early stopping was enabled during training to prevent overfitting.

**Figure 3:**
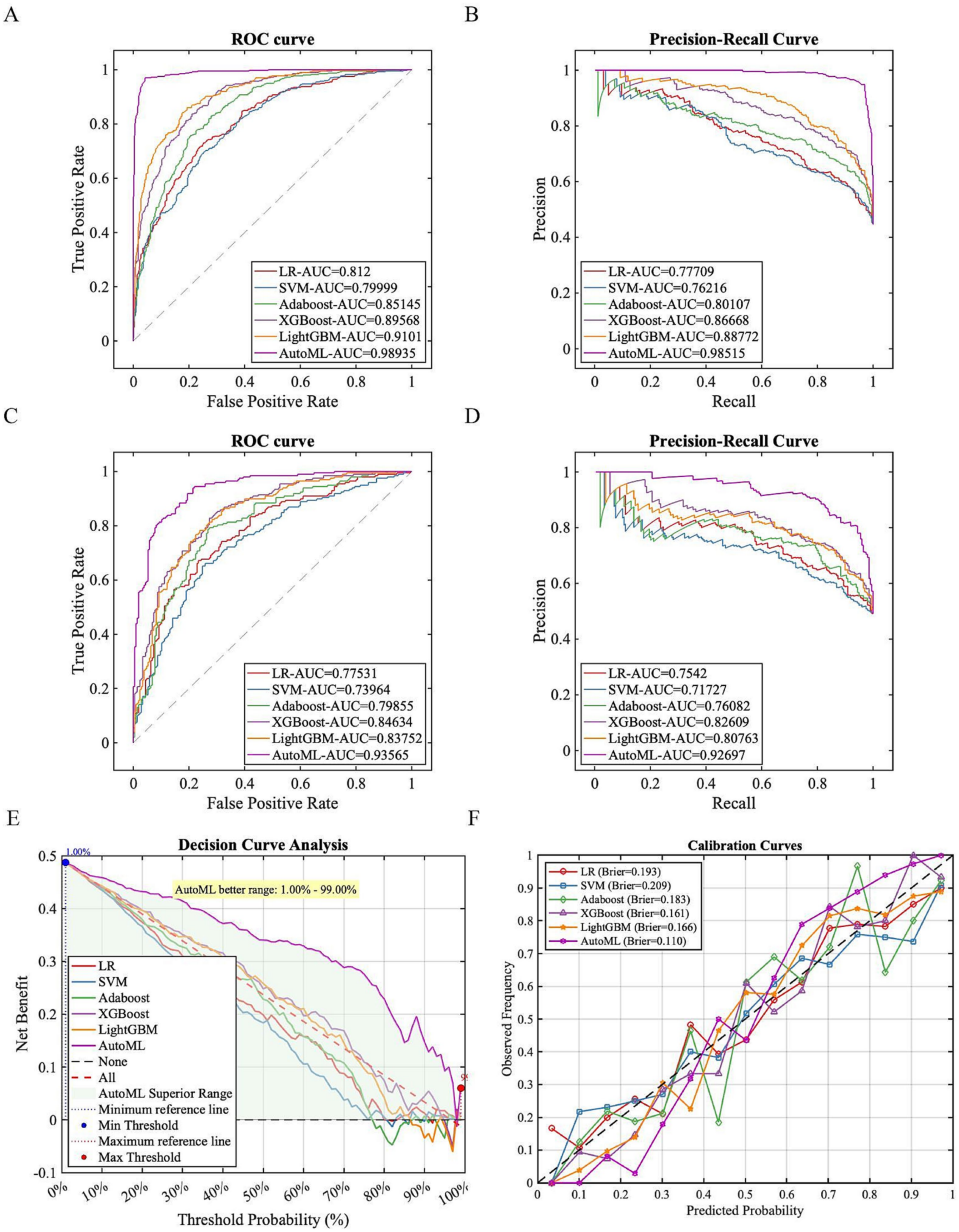
Model training and validation results. **(A)** Training set ROC curve; **(B)** training set PR curve; **(C)** test set ROC curve; **(D)** test set PR curve; **(E)** test set DCA curve, the horizontal axis of the curve represents the threshold probability, defined as the minimum mortality risk threshold at which clinicians determine intervention is warranted; the vertical axis represents the net benefit, which comprehensively accounts for both the benefit of true positives and the cost of false positives; **(F)** test set calibration curve.

Under this configuration, the discriminative performance of the six models on the training set is presented in Table 2. The AutoML model achieved best performance. The test set further confirmed its robustness, yielding a ROC-AUC of 0.9357 and PR-AUC of 0.9270 (Figures 3A–D). Decision curve analysis demonstrated that the AutoML model’s clinical net benefit surpassed that of traditional methods across a threshold range of 1–96% (Figure 3E); The calibration curve (Figure 3F) likewise indicated high consistency between predicted probabilities and actual outcomes, with a Brier score as low as 0.110.

**Table 2:** Performance evaluation metrics of predictive models.

Data set	Model	PPV	SEN	SPE	NPV	ACC	F1	ROC-AUC	PR-AUC
Training set	LR	0.5419	0.9375	0.3622	0.9282	0.6187	0.6868	0.8120	0.7771
	SVM	0.5398	0.9500	0.3481	0.9395	0.6165	0.6884	0.8000	0.7622
	AdaBoost	0.6629	0.8900	0.6358	0.9280	0.7492	0.7599	0.8515	0.8011
	XGBoost	0.6088	0.9725	0.4970	0.9758	0.7090	0.7488	0.8957	0.8667
	LightGBM	0.5942	**0.9775**	0.4628	0.9786	0.6923	0.7391	0.9101	0.8877
	AutoML	**0.8884**	0.9750	**0.9014**	**0.9877**	**0.9342**	**0.9297**	**0.9894**	**0.9852**
Testing set	LR	0.5344	0.9798	0.1756	0.9455	0.5707	0.6916	0.7753	0.7542
	SVM	0.5039	**0.9848**	0.0634	0.8926	0.5161	0.6667	0.7397	0.7173
	AdaBoost	0.6530	0.8838	0.5463	0.9034	0.7122	0.7511	0.7986	0.7608
	XGBoost	0.6944	0.8838	0.6244	0.9143	0.7519	0.7778	0.8463	0.8261
	LightGBM	0.6768	0.8990	0.5854	0.9202	0.7395	0.7722	0.8275	0.8076
	AutoML	**0.8053**	0.9192	**0.7854**	**0.9510**	**0.8511**	**0.8585**	**0.9357**	**0.9270**

Bold values indicate the best performance for each metric across all evaluated models.

### 3.4 Analysis of key predictive factors

**Figure 4:**
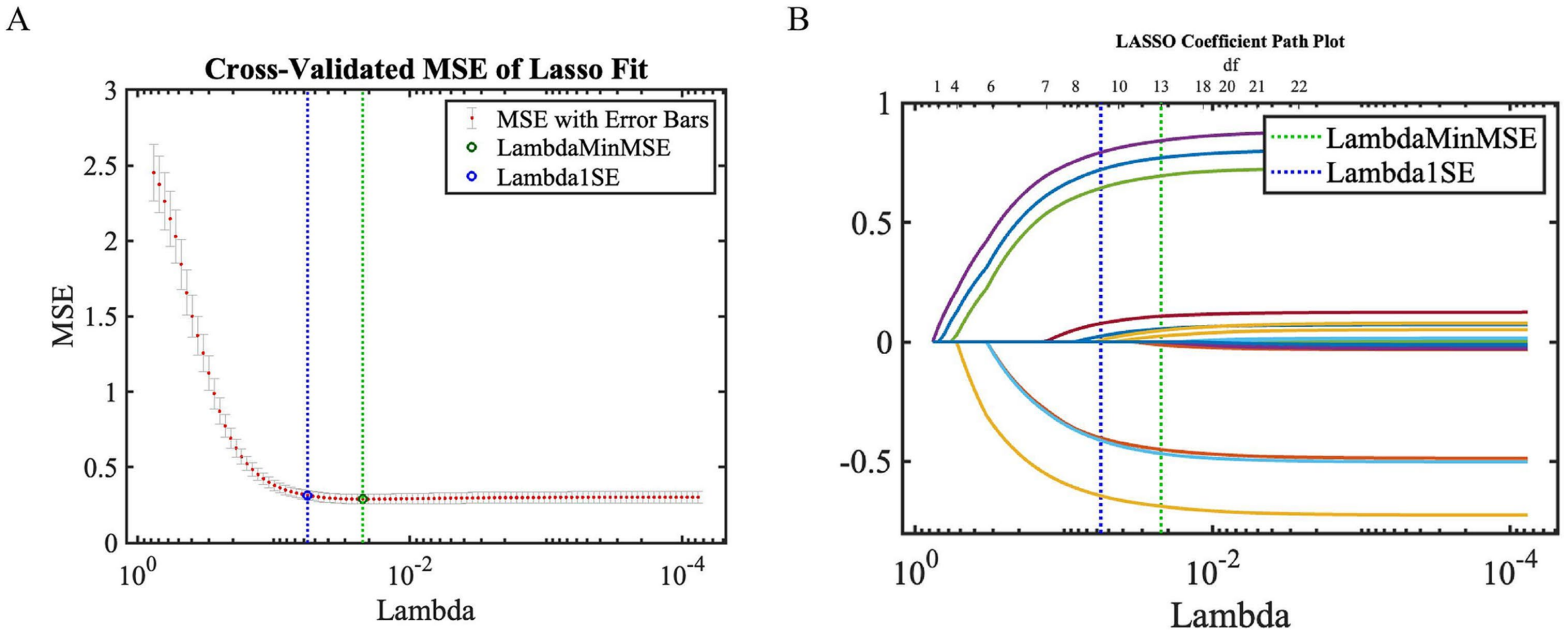
LASSO regression results. **(A)** Coefficient trajectory; **(B)** cross-validation fitting plot.

**Figure 5:**
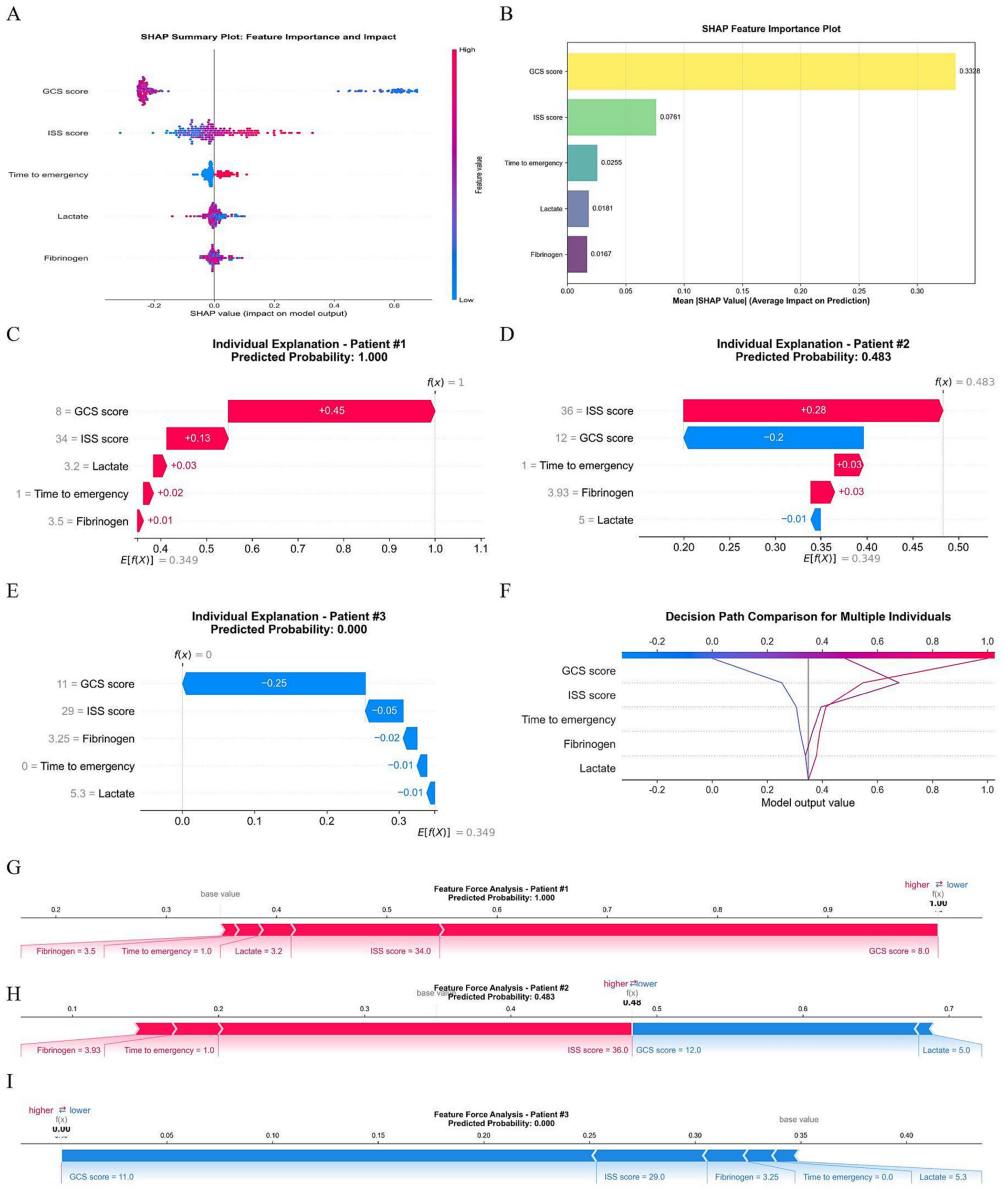
SHAP interpretability analysis. **(A)** The Shapley summary plot comprehensively presents the overall impact patterns of various features on model predictions across all samples. Each point in the plot represents a feature and its SHAP value (i.e., the feature’s contribution to prediction) for a specific sample. The color of the points indicates the actual value magnitude of the feature, while the distribution along the horizontal axis (SHAP values) reflects how feature values influence predictions (positive values increase predictions, negative values decrease them). This visualization allows intuitive identification of which features generally correlate with increases or decreases in predicted values, as well as trend relationships between feature influence and feature magnitudes; **(B)** the Shapley feature importance plot displays the overall importance ranking of each feature’s impact on model predictions in bar chart form. Feature importance is determined by calculating the mean absolute SHAP value for each feature across all samples, thereby measuring its average contribution to model output variations. Longer bars indicate greater influence of the feature in the model’s overall decision-making process, providing researchers with clear insight into the most critical factors driving predictions; **(C–E)** Waterfall plots illustrate the cumulative contribution process of each feature to individual patient predictions. The baseline value represents the model’s average prediction for all patients, while feature contributions show how each feature affects the final prediction (red indicating increased risk, blue indicating decreased risk). The sum of all feature contributions yields the final predicted value; **(F)** the decision path plot compares decision pathways across multiple patients, demonstrating how different feature combinations lead to varying prediction outcomes. The horizontal axis shows predicted probabilities, the vertical axis lists features, and the curved pathways trace decision routes from baseline values to final predictions; **(G–I)** force plots visually demonstrate how each feature “pushes” predictions toward higher or lower risk directions. Red arrows indicate features pushing predictions toward higher risk, blue arrows indicate features pushing toward lower risk, with arrow length representing the magnitude of influence.

(1) LASSO regression: LASSO regression validated AutoML-selected features (Figure 4). Using the Lambda1SE criterion (minimum MSE within one standard error), nine variables were retained, including all five AutoML features.

(2) SHAP analysis: feature importance ranked as: GCS > ISS > time from injury to ER > lactate > fibrinogen (Figures 5A,B). Decision paths (Figure 5F) revealed systematic shifts toward high-risk feature combinations in poor-prognosis patients.

Individual examples: high-risk patient (ID 1, Figures 5C,G): The predicted probability of poor prognosis for this patient is 100%. The patient has a GCS score of 8 (low score, high risk), an ISS score of 34 (relatively high), and a time from injury to ER admission >4 h—these factors collectively contribute to the poor prognosis. SHAP analysis shows the GCS score contributes most significantly (SHAP value = 0.4520), followed by the ISS score (SHAP value = 0.1347), indicating impaired consciousness and trauma severity are the primary determinants of prognosis. Despite normal lactate levels (3.20 mmol/L), the negative impacts of the low GCS and high ISS scores sufficiently lead to poor prognosis.

Moderate-risk patient (ID 2, Figures 5D,H): The predicted probability of poor prognosis is 48.3%, categorizing this patient as moderate-risk. The patient has a GCS score of 12 (relatively high, protective factor), an ISS score of 34 (relatively high, risk factor), and a time from injury to ER admission ≤4 h (protective factor). SHAP analysis reveals the GCS score as a protective factor (SHAP value = −0.2369) significantly reduces the risk of poor prognosis, while the ISS score as a risk factor (SHAP value = 0.0819) increases risk. This case demonstrates that even with a high ISS score, favorable consciousness (GCS = 12) and timely ER intervention (≤4 h) can partially mitigate poor prognosis risk, highlighting the importance of multifactorial assessment.

Low-risk patient (ID 3, Figures 5E,I): The predicted probability of poor prognosis is below 0.01%. The patient has a GCS score of 11 (relatively high, protective factor), an ISS score of 29 (moderate), and a time from injury to ER admission ≤4 h (protective factor)—all primary features exhibit protective effects. SHAP analysis indicates the GCS score is the strongest protective factor (SHAP value = −0.2532), substantially reducing risk, followed by the ISS score (SHAP value = −0.0524). This case confirms that patients with intact consciousness, moderate trauma severity, and prompt ER care generally have favorable outcomes, validating the model’s efficacy in identifying low-risk patients.

### 3.5 Clinical decision support system

**Figure 6:**
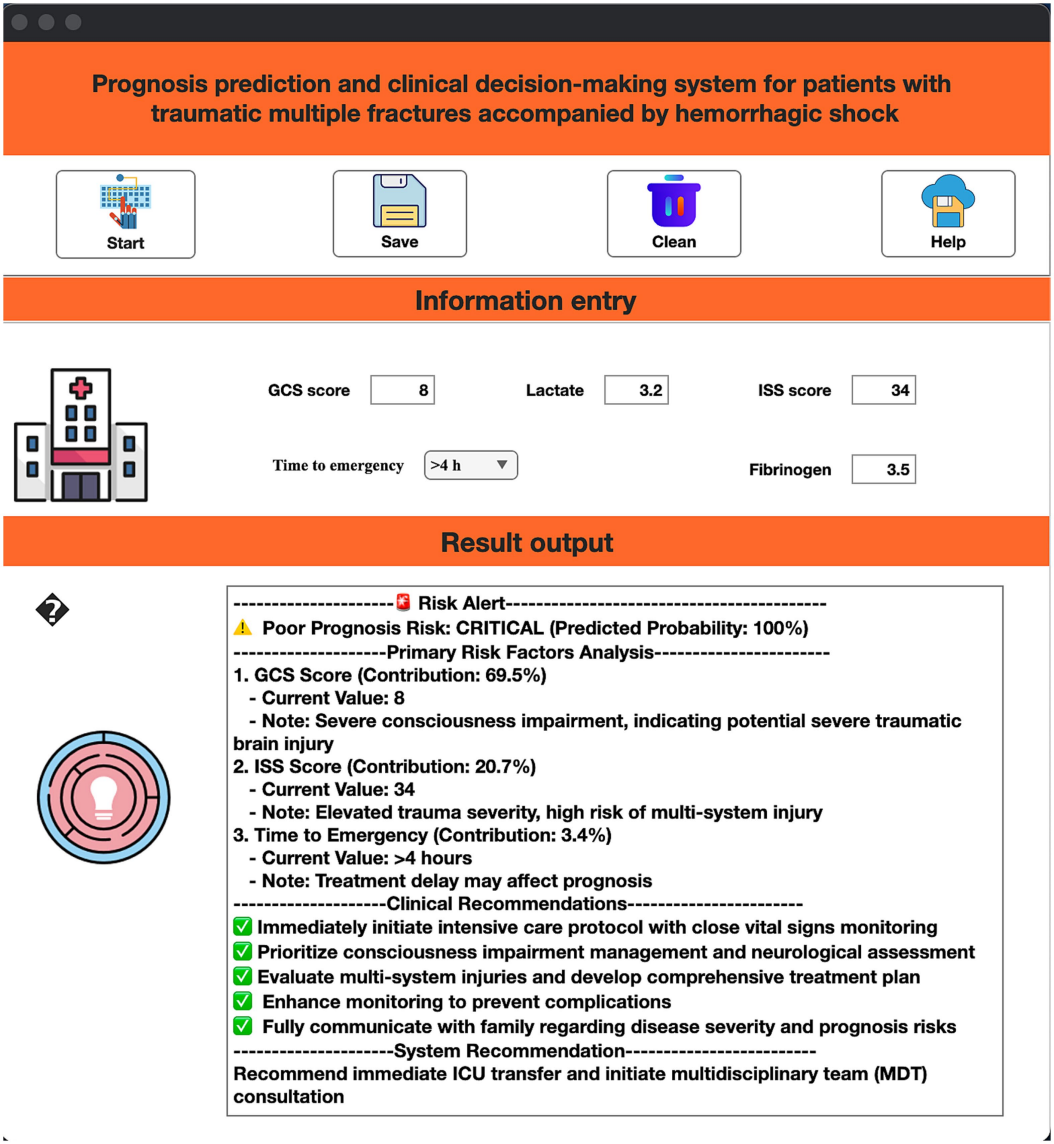
Software interface demonstration.

To overcome barriers in AI adoption (e.g., programming skills), an intuitive visualization system was developed using MATLAB 2024a App Designer. Clinicians input the five key features to instantly calculate mortality risk and receive care recommendations (Figure 6).

## 4 Discussion

Traumatic multiple fractures with hemorrhagic shock represent critical emergencies in emergency surgery and critical care medicine, combining the complexity of traumatic injuries with the high lethality of hemorrhagic shock. These patients face high in-hospital mortality risks, rapid disease progression, and multifactorial complexities, making accurate prognostic assessment and stratified management core challenges in clinical trauma resuscitation (22, 23). Since the prognosis of such patients is jointly influenced by multidimensional factors including trauma severity, systemic perfusion status, organ functional reserve, and timeliness of treatment, single indicators or traditional assessment methods often fail to comprehensively reflect patients’ pathophysiological status, easily leading to prognostic inaccuracies that compromise the scientific validity and timeliness of treatment decisions (24, 25).

This study developed an IBBO-based adaptive AutoML framework to construct an in-hospital mortality prediction model by integrating multisource clinical data encompassing demographic characteristics, trauma features, vital signs, and laboratory tests, while systematically comparing six machine learning models. The model demonstrated robust generalizability in independent testing, with a ROC-AUC of 0.9357 and a PR-AUC of 0.9270. Decision curve analysis indicated that the model could provide clinical net benefit across a wide risk threshold range of 1–96%, and calibration curves showed high consistency between predicted and observed mortality probabilities, yielding a Brier score of 0.110. Overall, these results suggest good discriminative ability, calibration performance, and potential clinical utility, without implying definitive superiority. Concurrently, LASSO regression validation and SHAP interpretability analysis identified GCS score, ISS score, injury-to-ER time, lactate level, and fibrinogen as core predictors of in-hospital mortality, providing key targets and scientific support for clinical identification of high-risk patients and targeted intervention strategies (26, 27).

SHAP analysis in this study revealed that the GCS score ranked first in importance among all predictive features, constituting the core determinant of mortality—a finding highly consistent with clinical trauma care principles (28). As a classical indicator of impaired consciousness, decreased GCS values directly reflect traumatic brain injury severity while indirectly indicating pathophysiological states of systemic hypoperfusion and cerebral hypoxia. Impaired consciousness represents both a common clinical manifestation and a critical marker of severity in these patients (29). The ISS score ranked second in importance, with higher scores indicating more severe multisystem injuries, intensified trauma-induced stress and inflammatory responses, and elevated bleeding risks. These collectively exacerbate hemorrhagic shock, leading to systemic perfusion disorders and multi-organ failure, explaining the significantly increased mortality observed in patients with ISS scores ≥34 (30, 31). Injury-to-ER time >4 h was identified as a high-risk factor, aligning with the “golden hour” and “golden 6 h” concepts in trauma care (32, 33), as delayed intervention allows progression from compensatory to decompensatory shock stages, causing irreversible tissue hypoperfusion and multi-organ dysfunction (34, 35). Elevated lactate signifies persistent tissue hypoxia driving anaerobic metabolism, while decreased fibrinogen indicates coagulopathy risk and potential disseminated intravascular coagulation (DIC), making both critical quantitative indicators for assessing severity and prognosis (36).

The superior performance of the IBBO-based AutoML model stems from its ability to manage high-dimensional data with complex nonlinear interactions inherent to this condition (37). Traditional models relying on manual feature engineering and hyperparameter tuning are vulnerable to clinical knowledge limitations and improper tuning (38). Our two-phase AutoML framework automates both processes: Phase I applies IBBO to select high-weight features in discrete space (e.g., GCS, ISS); Phase II refines hyperparameters in continuous space using 5-fold cross-validation and SMOTE for class balancing. Enhanced by chaotic mapping initialization and dynamic Lévy flight strategies, IBBO outperformed BBO, WOA, GWO, and PSO in convergence speed and global optimization capability. Furthermore, AutoML significantly reduces technical barriers, enabling rapid deployment without expert-level machine learning knowledge—crucial for clinical implementation (39, 40).

However, several important limitations should be carefully considered when interpreting our findings. First and most importantly, this study was a retrospective analysis, which may introduce selection bias and limit the generalizability of the results to other regions or patient populations. Accordingly, the conclusions should not be over-generalized, and prospective multicenter validation is essential before any broad clinical application can be contemplated. Second, we observed a notable performance gap between the training set (ROC-AUC: 0.9894) and the independent test set (ROC-AUC: 0.9357), which warrants careful interpretation. This discrepancy may stem from several factors. Inherent data heterogeneity between the retrospective training cohort and the temporally or geographically distinct test population likely introduced distributional shifts that challenged model generalization. Despite the application of L2 regularization (coefficient of 0.1) and early stopping, the exceptionally high training-set performance suggests that the model may have captured dataset-specific noise or idiosyncratic patterns, indicating a degree of overfitting that becomes more apparent when evaluated on unseen data. The relatively modest sample size of a retrospective study may have limited the model’s exposure to the full spectrum of trauma patient variability, making the learned decision boundaries somewhat brittle. To mitigate this gap in future work, we propose several directions: further strengthening regularization (e.g., increasing the L2 penalty or incorporating dropout strategies), expanding the dataset through multi-center prospective collection to enhance representativeness, and employing additional data augmentation or domain adaptation techniques to improve robustness against distributional shifts. Prospective external validation remains essential to confirm whether the observed performance can be sustained in broader clinical settings. Third, the model currently excludes dynamic treatment parameters (e.g., fluid resuscitation volume, surgical timing, vasopressor use), limiting its prognostic dimensionality and the ability to capture evolving clinical states. Fourth, while SHAP analysis provided valuable insights into feature importance, it may not fully resolve complex nonlinear interactions such as synergistic thresholds between GCS and ISS scores. Finally, the current MATLAB-based decision-support software lacks integration with Hospital Information Systems (HIS) or Electronic Medical Records (EMR), which hinders its clinical workflow efficiency. Future work should accordingly focus on: conducting multi-center, prospective studies to validate and potentially recalibrate the model across diverse healthcare settings; incorporating dynamic treatment indicators to enable real-time, time-series prognostic assessment; exploring advanced methods to capture nonlinear feature interactions and developing hybrid models that combine machine learning with explicit clinical rules; integrating the software into HIS/EMR environments and creating lightweight mobile or web-based versions for point-of-care use; and designing targeted intervention protocols for high-risk subgroups (e.g., those with low GCS and high ISS) to prospectively evaluate whether model-guided stratified management can improve clinical outcomes.

## 5 Conclusion

In summary, this IBBO-based AutoML model provides an efficient tool for mortality risk assessment in patients with traumatic multiple fractures and hemorrhagic shock. Core predictors—GCS score, ISS score, injury-to-ER time, lactate, and fibrinogen—combined with the clinical decision-support software, offer visualized and intelligent stratification guidance, making it a promising adjunct in trauma resuscitation practice.

## Data Availability

The raw data supporting the conclusions of this article will be made available by the authors, without undue reservation.
