# Supplementary material for: Development and validation of a prognostic prediction model for patients with traumatic multiple fractures and hemorrhagic shock using an Automated Machine Learning framework: a retrospective cohort study
Source: Front Med (Lausanne). 2026 Jun 10;13:1837872. doi: 10.3389/fmed.2026.1837872 (PMC13292078; doi:10.3389/fmed.2026.1837872)
Supplement: Supplementary file 1 [file Table_1.DOCX]

**1.Summary of variables collected for model development**

All variables used for model training were summarized, and the data types of each variable were labeled one by one according to four categories: demographic and baseline characteristics, trauma/injury characteristics, admission vital signs and perfusion indicators, and laboratory parameters.

**Table S1. Summary of variables collected for model development**

| Category | Variable | Type | Missing (%) |
| --- | --- | --- | --- |
| Demographics and baseline characteristics | Age | Continuous | 0 |
|  | Sex | Categorical | 0 |
|  | Body mass index (BMI) | Continuous | 1.2 |
|  | Comorbidities | Categorical | 0.5 |
| Trauma and injury characteristics | Injury mechanism | Categorical | 0 |
|  | Time from injury to ER admission | Categorical | 2.1 |
|  | Traumatic brain injury | Categorical | 0 |
|  | Abdominal solid organ injury | Categorical | 0.3 |
|  | Injury Severity Score (ISS) | Continuous | 0.8 |
|  | Glasgow Coma Scale (GCS) | Continuous | 0.5 |
| Admission vital signs and perfusion indices | Systolic blood pressure | Continuous | 0 |
|  | Diastolic blood pressure | Continuous | 0 |
|  | Heart rate | Continuous | 0 |
|  | Respiratory rate | Continuous | 1.8 |
|  | Temperature | Continuous | 0.4 |
|  | Shock index | Continuous | 0 |
| Laboratory parameters | Hemoglobin (Hb) | Continuous | 0.2 |
|  | Hematocrit (HCT) | Continuous | 0.3 |
|  | Platelet count (PLT) | Continuous | 0.1 |
|  | Fibrinogen (Fib) | Continuous | 1.5 |
|  | Activated partial thromboplastin time (APTT) | Continuous | 0.7 |
|  | Prothrombin time (PT) | Continuous | 0.6 |
|  | International normalized ratio (INR) | Continuous | 1.0 |
|  | Arterial lactate (Lac) | Continuous | 1.2 |
|  | Base excess (BE) | Continuous | 1.4 |
|  | Creatinine (Cr) | Continuous | 0.3 |
|  | Blood urea nitrogen (BUN) | Continuous | 0.5 |
|  | Alanine aminotransferase (ALT) | Continuous | 0.9 |
|  | Sodium | Continuous | 0.1 |
|  | Potassium | Continuous | 0.1 |

**2. SMOTE resampling quantization gain result**

To quantify the actual benefit of SMOTE resampling for the model adopted in this study, we compared the performance of the AutoML model on the test set under two conditions—with and without SMOTE—using the same data partition, feature subset, and hyperparameter configuration. As shown in Table 3, without SMOTE, the AutoML model achieved a positive predictive value (PPV) of 0.7135, sensitivity of 0.7192, specificity of 0.7854, negative predictive value (NPV) of 0.8829, accuracy of 0.7614, F1 score of 0.7511, ROC-AUC of 0.8912, and PR-AUC of 0.8845 on the test set, indicating insufficient ability to identify the minority class under the original imbalanced data. After applying SMOTE to rebalance the training set, the PPV increased to 0.8053, sensitivity improved substantially to 0.9192, specificity remained at 0.7854, NPV reached 0.9510, accuracy rose to 0.8511, F1 score increased to 0.8585, ROC-AUC significantly improved to 0.9357, and PR-AUC reached 0.9270. These results demonstrate that the SMOTE resampling strategy markedly enhanced the model’s ability to detect deceased cases without compromising specificity, and the precision-recall balance was substantially improved, fully validating the necessity and effectiveness of applying SMOTE to address class imbalance in this study.

**Table S2. Effect of SMOTE resampling on the test set performance of the AutoML model**

| Resampling strategy | PPV | SEN | SPE | NPV | ACC | F1 | ROC-AUC | PR-AUC |
| --- | --- | --- | --- | --- | --- | --- | --- | --- |
| Without SMOTE | 0.7135 | 0.7192 | 0.7854 | 0.8829 | 0.7614 | 0.7511 | 0.8912 | 0.8845 |
| With SMOTE | 0.8053 | 0.9192 | 0.7854 | 0.9510 | 0.8511 | 0.8585 | 0.9357 | 0.9270 |
